# Supplementary figures and images for: Evaluation of colonization and mutualistic endophytic symbiosis of Escherichia coli with tomato and Bermuda grass seedlings
Source: PeerJ. 2022 Aug 10;10:e13879. doi: 10.7717/peerj.13879 (PMC9375544; doi:10.7717/peerj.13879)

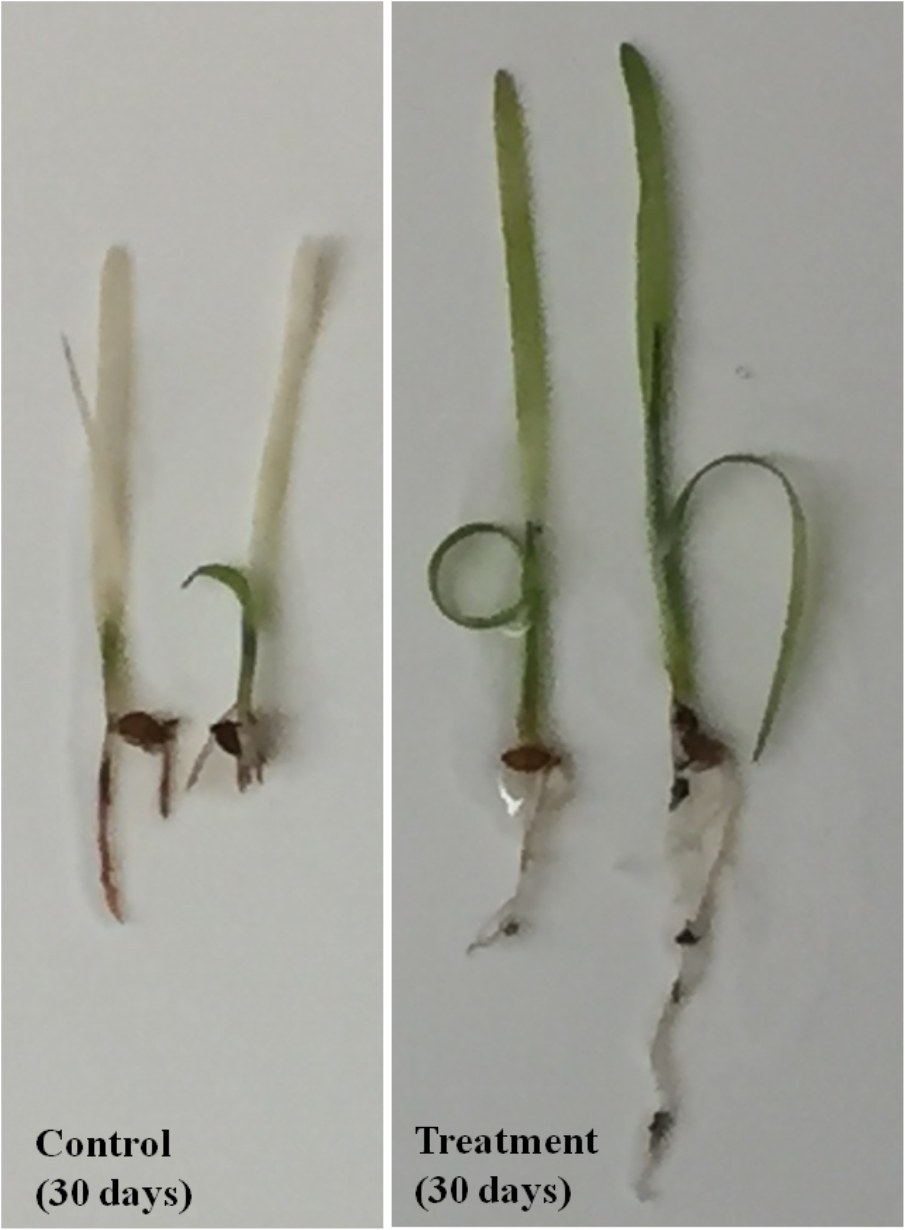

Supplement: Supplemental Information 1 [file peerj-10-13879-s001.png]
