# Supplementary material for: Evaluation of colonization and mutualistic endophytic symbiosis of Escherichia coli with tomato and Bermuda grass seedlings
Source: PeerJ. 2022 Aug 10;10:e13879. doi: 10.7717/peerj.13879 (PMC9375544; doi:10.7717/peerj.13879)
Supplement: Supplemental Information 2 — Supplementary Table 1. IAA (auxin) production by E. coli (GFP) in LB broth medium with and without tryptophan, antifungal activity against F. oxysporum, Curvularia sp. and Alternaria sp., and phosphate solubilisation. Supplementary Table 2. Initial screening of plant growth promoting features of E. coli (GFP) on Bermuda onto agarose and MS- agarose media. [file peerj-10-13879-s002.docx]

**Supplementary Table 1.** IAA (auxin) production by *E. coli* (GFP) in LB broth medium with and without tryptophan, antifungal activity against *F. oxysporum, Curvularia* sp*.* and *Alternaria* sp., and phosphate solubilization.

| **IAA (Auxin)**  **µg ml^-1^** | | **Antifungal activity**  **(% inhibition)** | | | **Phosphate solubilization** |
| --- | --- | --- | --- | --- | --- |
| With tryptophan | Without tryptophan | *F. oxysporum* | *Curvularia* sp. | *Alternaria* sp. |  |
| 8.68±0.43 |  | 13% | 29% | 13% | None |

**Supplementary Table 2.** Initial screening of plant growth promoting features of *E. coli* (GFP) on Bermuda onto agarose and MS- agarose media.

| Treatments | Growth Promoting Features | | | |
| --- | --- | --- | --- | --- |
|  | Germination  (%) | Geotropic response (%) | Root –Shoot length (cm) | Root hairs |
| Control-Agarose | 16.67 | 10 | R=1.0-1.5  S= 1.0-1.5 | many and short |
| Control-MS | 33.33 | 85 | R=1.5-2.0  S= 1.5-2.0 | very few and short |
| Treatment-Agarose | 28.33 | 88.23 | R=2.0-2.5  S= 1.5-2.0 | more abundant and long |
| Treatment-MS | 30 | 100 | R=1.5-2.0  S= 1.5-2.0 | more abundant and long |
